# STARD3NL inhibits the osteogenic differentiation by inactivating the Wnt/β‐catenin pathway via binding to Annexin A2 in osteoporosis

**DOI:** 10.1111/jcmm.17205

**Published:** 2022-01-30

**Authors:** Yuexin Xu, Xiaogang Bao, Xiaoyun Chen, Peixuan Wu, Shiyu Chen, Bowen Zhang, Jing Ma, Guohua Xu, Duan Ma

**Affiliations:** ^1^ Department of Biochemistry and Molecular Biology School of Basic Medical Sciences Fudan University Shanghai China; ^2^ Department of Orthopedic Surgery The Spine Surgical Center Second Affiliated Hospital of Naval Medical University Shanghai China; ^3^ Li Ka Shing Faculty of Medicine The University of Hong Kong Hong Kong City Hong Kong; ^4^ Department of Facial Plastic and Reconstructive Surgery ENT Institute Eye & ENT Hospital Fudan University Shanghai China

**Keywords:** Annexin A2, osteogenic differentiation, Stard3nl, Wnt/β‐catenin

## Abstract

Osteoporosis is one of the leading forms of systemic diseases related to bone metabolism in the world. STARD3 N‐terminal like (STARD3NL) showed robust association with osteoporosis‐related traits. Yet, the molecular functional mechanisms of STARD3NL in osteoblasts is still obscure. In this study, we demonstrated a high level of STARD3NL expression in the bone tissues from the patients with low bone mass and ovariectomized (OVX)‐induced osteoporotic mice. We identified Stard3nl as a potent factor that negatively and positively regulates osteoblast differentiation and cell proliferation, respectively. Furthermore, inhibition of Stard3nl induced β‐catenin gene expression and the nuclear translocation of β‐catenin, as well as Wnt signalling activities, contributing to the activation of Wnt/β‐catenin signalling. Mechanistic studies revealed that Stard3nl bound with Annexin A2 (Anxa2) to suppress β‐catenin expression, resulting into the suppression of Wnt signalling and downstream osteogenic differentiation. Moreover, adeno‐associated virus 9 (AAV9)‐mediated silencing of Stard3nl reversed bone loss in OVX‐induced osteoporotic mice by the injection into the knee joints. Collectively, our study revealed that Stard3nl suppressed osteogenesis via binding with Anxa2, resulting into the inactivation of Wnt signalling. It also highlights the preventive and therapeutic potential of STARD3NL as a specific and novel target for osteoporotic patients.

## 1 INTRODUCTION

As a highly prevalent disorder, osteoporosis is an emerging socioeconomic threat that imposes an overwhelming financial burden by its concomitant fragility fractures; the prevalence of osteoporosis is foreseen to increase as the population ages.^1^ Osteoporosis is caused by the imbalance of bone homeostasis, which is preserved by osteoblasts and osteoclasts that are bone‐forming activity and bone‐resorptive activity, respectively.^2^ Osteoblast‐mediated bone formation arises out of the primitive skeletal stem cells; These stem cells further differentiate into osteoblast precursors, which undergo maturation to generate osteoblasts, and finally, trigger the formation of the bone matrix and initiate mineralization through further differentiation.^3^ Osteoblast dysfunction breaks the balance, tipping the scale towards bone resorption mediated by osteoclast while against osteoblastic bone formation, resulting in pathological bone destruction and loss.^4^ By employing genome‐wide association studies (GWAS) in the field of bone health, studies have devoted tremendous efforts to identifying more than 500 osteoporosis‐susceptibility loci predominantly by examining millions of single nucleotide polymorphisms (SNPs).^5^ However, the causal genetic variants in disease‐associated genes and the underlying mechanisms have greatly been overlooked.

StAR‐related lipid transfer domain containing 3 N‐terminal like (STARD3NL, also known as MENTHO) is a STARD3 paralogue without the START domain.^6^ STARD3NL localizes predominantly at the late‐endosomal membranes, and at lysosomal membranes and other organelle contact sites.^7^, ^8^, ^9^ Later identified as a late endosome‐resident protein, Stard3nl mediates the endosomal cholesterol transport through the MENTAL domain.^10^ Through an FFAT‐like motif located in the MENTAL domain, Stard3nl can interact with VAP proteins to create endosome–endoplasmic reticulum (ER) contacts, which provide a favourable environment for the exchange of different molecules and specific metabolic activities.^11^ Emerging evidence has shown that SNPs associated with the STARD3NL are linked to traits related osteoporosis.^5^, ^12^, ^13^, ^14^, ^15^ Yet, the direct functional mechanism of STARD3NL in osteoporosis remains unclear.

The Wnt signalling transduction cascade was found an important modulator in embryogenesis, carcinogenesis and the development of many growth‐related pathologies.^16^ Wnt signalling pathways have two major types, one being canonical and dependent of β‐catenin, the other being non‐canonical and independent of β‐catenin. The ‘canonical’ β‐catenin‐dependent pathway involves extracellular Wnt ligand binding to lipoprotein receptor‐related protein (LRP)5/6 and FZD receptors‐induced stabilization and translocation of β‐catenin to the nucleus where it interacts with the LEF/TCF transcription factors to modify target gene transcription.^17^, ^18^ Emerging evidence has demonstrated that the former one, canonical Wnt signalling pathway, involves greatly in mediating the bone homeostasis and pathogenesis of various bone‐related disorders.^19^

In this study, we examined and analysed the expression of STARD3NL in spinous processes of healthy control and patients with low bone mass. We demonstrated that enforced expression of Stard3nl in a murine osteoblastic cell line causes increase in cell proliferation and migration and decrease in β‐catenin expression, ALP activity and bone mineralization. Mechanistically, the negative regulator of osteogenic differentiation mediated this process by binding with Anxa2 via the Wnt signalling. Our data suggest a novel mechanistic insight into the association of Stard3nl with osteogenic differentiation and also reveal a promising therapeutic target for osteoporosis.

## 2 MATERIALS AND METHODS

### 2.1 Patients and clinical bone specimens

Altogether, 34 spinous processes were obtained from patients with low bone mass (19 patients with osteopenia and 15 patients with osteoporosis) or healthy controls who underwent spinal operation (50–80 years old) referred to Second Affiliated Hospital of Naval Medical University. Approvals were obtained from Human Research Ethics Committee of Second Affiliated Hospital of Naval Medical University (no. 2017SL040). The osteoporosis group with a BMD T‐scores ≤ −2.5 standard deviation (SD), the osteopenia group with a BMD T‐scores between −1.0 and −2.5 and the control group with a T‐scores ≥ −1.0 SD were determined by DXA. Written informed consent of each subject was received prior to specimen collections. Bone tissues were prepared in liquid nitrogen until further use.

### 2.2 Cell line, cell culture and osteogenic differentiation

Murine embryonic mesenchymal stem cell line C3H10T1/2 and pre‐osteoblast MC3T3‐E1 (Cell Bank of the Chinese Academy of Science; Shanghai, China) were maintained in MEM and α‐MEM (Gibco, Thermo Fisher Scientific, Inc.), respectively. 293T cells from American Type Culture Collection (ATCC, Manassas, VA) and mouse myoblast cell line C2C12 from Cell Bank of the Chinese Academy of Science were cultured in DMEM at 37˚C in a humidified atmosphere containing 5% CO_2_, commonly with 10% foetal bovine serum (FBS; Sigma‐Aldrich, Louis, MO, USA). All these dishes and plates for cell culture were purchased from in vitro scientific (Hangzhou Xinyou Biotechnology; Hangzhou, China). Cells were seeded in the standard growth medium and incubated overnight to adhere. Next, cells were incubated with BMP2 (50 ng/ml) (Proteintech, Rosemont, USA), with an exchange of fresh BMP2 every two days.

### 2.3 Animals and AAV9‐mediated delivery in OVX‐induced osteoporosis

Procedures related to mice and the corresponding experimental protocols were approved by the Institutional Animal Care and Use Committee of Fudan University. The standard animal facility provided free access to water and food to accommodate these mice (12‐h light/dark cycle) at the animal centre of Fudan University. These tissues from the euthanized C57BL/6 mice by cervical dislocation underwent immediate dissection followed by immersion into liquid nitrogen separately.

To mimic oestrogen deficiency‐induced osteoporosis, these female C57BL/6 mice were subjected to bilateral OVX or sham surgery at 8 weeks of age. After seven weeks, these mice were injected into knee joints with AAV9 (1 × 10^11^ GC; 5 × 10^12^ GC/kg; OBiO Technology Corp., Ltd. Shanghai, China) carrying control (Ctrl) or Stard3nl shRNA (ShStard3nl) (n=5/group). Six weeks after injection, the mice were euthanized and the distal femur was collected for micro‐CT scanning and RNA extraction. Micro‐CT analysis was performed as described previously.^20^

### 2.4 RNA and reverse transcription‐quantitative real‐time PCR (RT‐qPCR)

By utilizing TRIZOL Reagent (Invitrogen, Thermo Fisher Scientific, Inc.), total RNA was isolated following the protocol from the manufacturer. PrimeScript RT reagent kits with gDNA Eraser (Takara Biotechnology Co., Ltd.) was used for synthesis of cDNA in reverse transcription reactions. PCR amplification was performed by TB Green reagents (Takara) on the ABI StepOne instrument (Applied Biosystems; Thermo Fisher Scientific, Inc.). Gapdh was employed as an internal normalization control. The PCR primers used sequences were as stated below: STARD3NL, forward, 5′‐TTGGTGGGCAATAGCGTTGA‐3′ and reverse, 5′‐GCAGCACATAGCCAAAAGCC‐3′; GAPDH, forward, 5′‐GGAGCGAGATCCCTCCAAAAT‐3′ and reverse, 5′‐GGCTGTTGTCATACTTCTCATGG‐3′; Stard3nl, forward, 5′‐TTGAGTCCTATGAAGGAAGGGAA‐3′ and reverse, 5′‐GCCTCCGTTCACATTTAACTCTA‐3′; Runt‐related transcription factor 2 (Runx2), forward, 5′‐GACTGTGGTTACCGTCATGGC‐3′ and reverse, 5′‐ACTTGGTTTTTCATAACAGCGGA‐3′; Osterix (Sp7), forward, 5′‐GGAAAGGAGGCACAAAGAAGC‐3′ and reverse, 5′‐CCCCTTAGGCACTAGGAGC‐3′; Alkaline phosphatase (Alpl), forward, 5′‐CCAACTCTTTTGTGCCAGAGA‐3′ and reverse, 5′‐GGCTACATTGGTGTTGAGCTTTT‐3′; Osteocalcin (Ocn), forward, 5′‐TGCTTGTGACGAGCTATCAG‐3′ and reverse, 5′‐GAGGACAGGGAGGATCAAGT‐3′; Bone sialoprotein (Ibsp), forward, 5′‐ATGGAGACGGCGATAGTTCC‐3′ and reverse, 5′‐CTAGCTGTTACACCCGAGAGT‐3′; Type I collagen α (Col1α1), forward, 5′‐GCAACAGTCGCTTCACCTACA‐3′ and reverse, 5′‐CAATGTCCAAGGGAGCCACAT‐3′, and Gapdh, forward, 5′‐AGGTCGGTGTGAACGGATTTG‐3′ and reverse, 5′‐TGTAGACCATGTAGTTGAGGTCA‐3′.

### 2.5 Western blot assay and Immunoprecipitation

Lysates were performed using RIPA buffer (Thermo Fisher Scientific, Inc.) plus protease inhibitors on ice. These denatured proteins were separated via 10% SDS‐PAGE and then transblotted onto nitrocellulose membrane. Non‐specific protein–protein interactions were blocked, followed by probing using the indicated antibody overnight at 4˚C. Then, the blot was conducted with secondary antibodies and finally visualized with ECL reagent. Primary antibodies against Stard3nl, Anxa2, Ibsp, Flag, β‐catenin (Proteintech, Rosemont, USA) and Alpl (Abcam, Cambridge, UK) were diluted at 1:1,000. Gapdh (1:5,000; Abcam) served as loading controls.

For immunoprecipitation experiments, lysates were pulled down with antibodies for 4 h and then rotated with protein A/G beads (Thermo Fisher Scientific) overnight at 4 °C. For Flag immunoprecipitation, anti‐Tag Mouse Antibody (agarose Conjugated) beads were directly incubated and rotated with cell lysates overnight at 4 °C. Bound proteins on beads were eluted in 2× SDS loading buffer for 10‐min boiling after washing three times with RIPA.

### 2.6 Construction of stably transfected cell lines by lentivirus transfection

Stard3nl shRNA plasmids were constructed with the pLKO.1‐puro‐vector. The Stard3nl‐Sh1 target was 5′‐CCAGTGCCTTTCTATTAGCAA‐3′; the Stard3nl‐Sh2 target was 5′‐CGATTCAAAGTGCTGATACTT‐3′; Stard3nl overexpression plasmid was cloned into a PCDH vector by Generay Biotech (Shanghai, China). A vector with no insert was used as a control. Briefly, 293T cells were applied for lentivirus packaging with L‐PEI, and 48 h post‐transfection the viral supernatants were obtained. Following the viral infection twice in cultured cells, stably transfected cells were acquired through at least 14 days of puromycin (10 µg/ml) screening. The efficiency of cell transfection was examined with Western blot and RT‐qPCR.

### 2.7 Cell proliferation assay

Cell proliferation abilities were first examined adopting the Cell Counting Kit‐8 (CCK‐8) assay (Shanghai Yeasen Biotechnology, Shanghai, China). Cells were seeded in 96‐well plates with growth medium for 4 days, and 10 µl of CCK‐8 solution was added to the corresponding well. After incubation of another 1 h, the optical density (OD) was recorded at a wavelength of 450 nm using a microplate reader. The EdU assays (RiboBio Co., Guangzhou, China) were also applied to examine cell proliferation following the protocol from the manufacturer. Incubation of cultured cells was conducted in 96‐well plates at an EdU concentration of 50 µM. Following Apollo Stain and permeabilization, cultured cells stained with Hoechst Stain were visualized under an EVOS^TM^ Microscope M5000 Imaging System (Invitrogen).

### 2.8 Transwell migration assay and Wound‐healing assay

Cells were cultured without FBS in the upper chamber, and 20% FBS medium was added to the lower chamber of the 8‐µm micropore inserts in 24‐well plates for the transwell migration assay. After incubating 24 h, the migrated cells were fixed and stained using 0.5% crystal violet. Microscope (Invitrogen) was employed to capture the images, and the number of the migrated cells was randomly counted by Image J software. Cell migration was again detected by the wound healing. The seeded cells in the 6‐well cell plates were allowed to attach overnight. Till these cells were reached near 100% confluency, 200‐μl sterile pipette tips were used to create a gap. The wounded closures were visualized using microscope at 0 h and 12 h.

### 2.9 ALP staining and Alizarin Red S staining

After incubation of 4–5 days, cultured cells underwent fixation with 95% ethanol and air‐dried, and then stained with ALP staining kit using the standard protocol (Beyotime). After rinsing with distilled water, the ALP‐positive cells were demonstrated in bluish or purplish staining. After osteogenic induction of 7–12 days, detection of the mineral deposition of osteoblast was conducted. Followed by fixation with 95% ethanol, freshly prepared 2% ARS solution was added to the cells (Sigma‐Aldrich). Subsequently, the dye in excess was washed with distilled water. Positive cells were stained cells as red colour and imaged using the iPhone 7 camera.

### 2.10 Immunofluorescence

Cell lines plated in 96‐well plates underwent fixation with 4% PFA, followed by 20‐min permeabilization. After blocking with 0.5% FBS, anti‐Stard3nl, anti‐Anxa2 and anti‐β‐catenin antibody were added into cells with dilutions at 1:100, 1:350 and 1: 250 with antibody diluent, respectively, and incubated overnight at 4 °C and followed by the fluorescent secondary antibodies. After stained with 1:1000 diluted DAPI, photographs were taken using microscope.

### 2.11 TOPFlash reporter assay

A TOPFlash reporter assay was performed to examine the role which Stard3nl expression played on the Wnt/β‐catenin signalling activities. After plated on 96‐well plates, stably transfected cells underwent transient transfection using the TOPFlash luciferase reporter plasmid, as well as the Renilla plasmid. Renilla luciferase activity was assessed as the internal control for normalization of the firefly luciferase activity.

### 2.12 mRNA sequencing

The C3H10T1/2 cells stable expressing Stard3nl or vector were prepared. Total RNA extraction was performed by TRIZOL Reagent, followed by the quantification and qualification of these RNA. Library preparations of next‐generation sequencing were performed and then libraries multiplexed with different indices were loaded on the Illumina HiSeq instrument following the manufacturer's protocol. An Illumina HiSeq (Illumina, San Diego, CA, USA) was then employed for the transcriptome sequencing, which was then processed and analysed by GENEWIZ (Suzhou, China). Genes were considered to filter out with P‐value >0.01 and log_2_ (fold change) <0.5, and the data were employed to GO/KEGG analysis.

### 2.13 Liquid chromatography–mass spectrometry

Protein identification was performed by mass spectrometry (Shanghai Applied Protein Technology Co., Ltd., Shanghai, China). A Q Exactive (Thermo Fisher) was employed to conduct the liquid chromatography–mass spectrometry (LC‐MS) analysis. Easy‐nLC 1000 (Thermo Fisher) system was then applied for the MS analysis. MASCOT software was used to analyse data and identify proteins bound to Stard3nl.

### 2.14 Statistical analysis

Experimental data are denoted as mean ±standard deviation. These results were analysed using Prism 6.0 software (GraphPad Software, La Jolla, CA, USA). Differences between means of independent groups were assessed by Student's t‐test or one‐way analysis of variance with Bonferroni's correction. In all experiments, differences with P‐value <0.05 were defined as statistically significant.

## 3 RESULTS

### 3.1 Alterations of STARD3NL expression in osteoporosis

**FIGURE 1:**
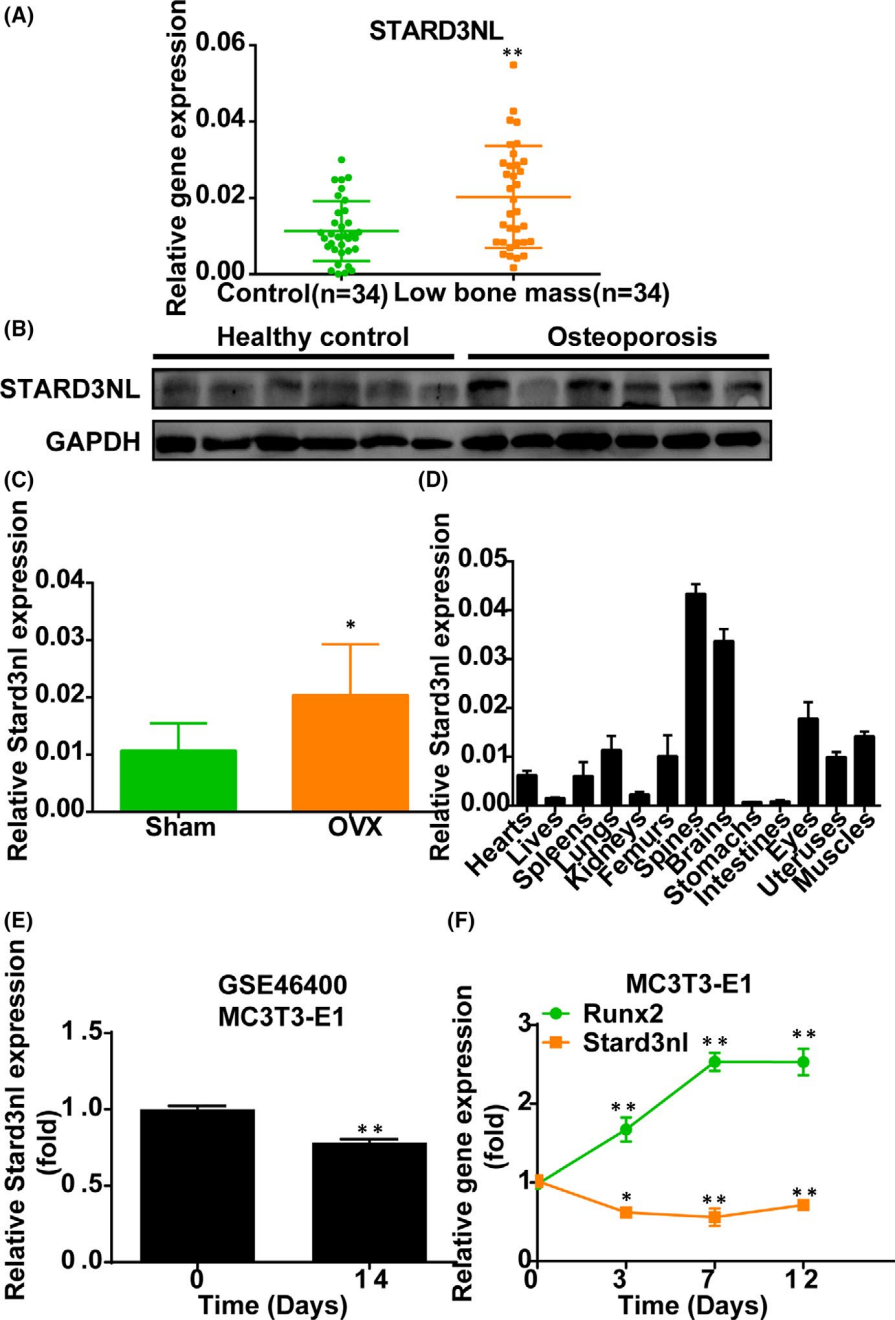
Expression levels of STARD3NL in osteoporosis. (A) RT‐qPCR of STARD3NL expression in humans with low bone mass. (B) Western blot of STARD3NL expression in humans with osteoporosis. (C) RT‐qPCR of Stard3nl expression in OVX‐induced mice. (D) The mRNA levels of Stard3nl in 13 types of tissues. (E) Bioinformatics analysis of STARD3NL mRNA expression based on the GEO datasets (GSE46400). (F) The mRNA levels of Stard3nl in MC3T3‐E1 cells during osteogenic differentiation. *p < 0.05 and **p < 0.01

To take a tentative step towards exploring the function of STARD3NL in osteoporosis, we detected the STARD3NL level in the bone tissues from human with low bone mass. Notably, the elevated level of STARD3NL was consistently observed in human with low bone mass relative to healthy controls (Figure 1A,B). When compared to the sham group, the upregulation of Stard3nl expression in the bone tissues from OVX‐induced mice was further confirmed by RT‐qPCR (Figure 1C).

For further functional evaluation of Stard3nl, tissue distribution analysis was conducted using the RT‐qPCR in normal adult mice. Detectable Stard3nl expression levels were observed in all 13 types of tissues, including the hearts, the livers, the spleens, the lungs, the kidneys, the femurs, the spines, the brains, the stomachs, the intestines, the eyes, the uteruses and the muscles. Moreover, results demonstrated preferential expressions of Stard3nl in the spine and the brain (Figure 1D).

Gene expression analysis was adopted for further exploration of potential functions of Stard3nl in osteogenesis by utilizing GEO datasets GSE46400, which exhibited the lower expression of Stard3nl after osteogenic induction for 14 days in MC3T3‐E1 cells (Figure 1E). Furthermore, RT‐qPCR confirmed that the Stard3nl was notably downregulated during the osteogenic differentiation phase in MC3T3‐E1 cells, while Runx2 increased gradually during the osteogenic differentiation phase (Figure 1F). Together, these findings suggest that Stard3nl may modulate osteogenic differentiation.

### 3.2 Stard3nl overexpression promotes cell growth and migration of C3H10T1/2 and C2C12 cells

**FIGURE 2:**
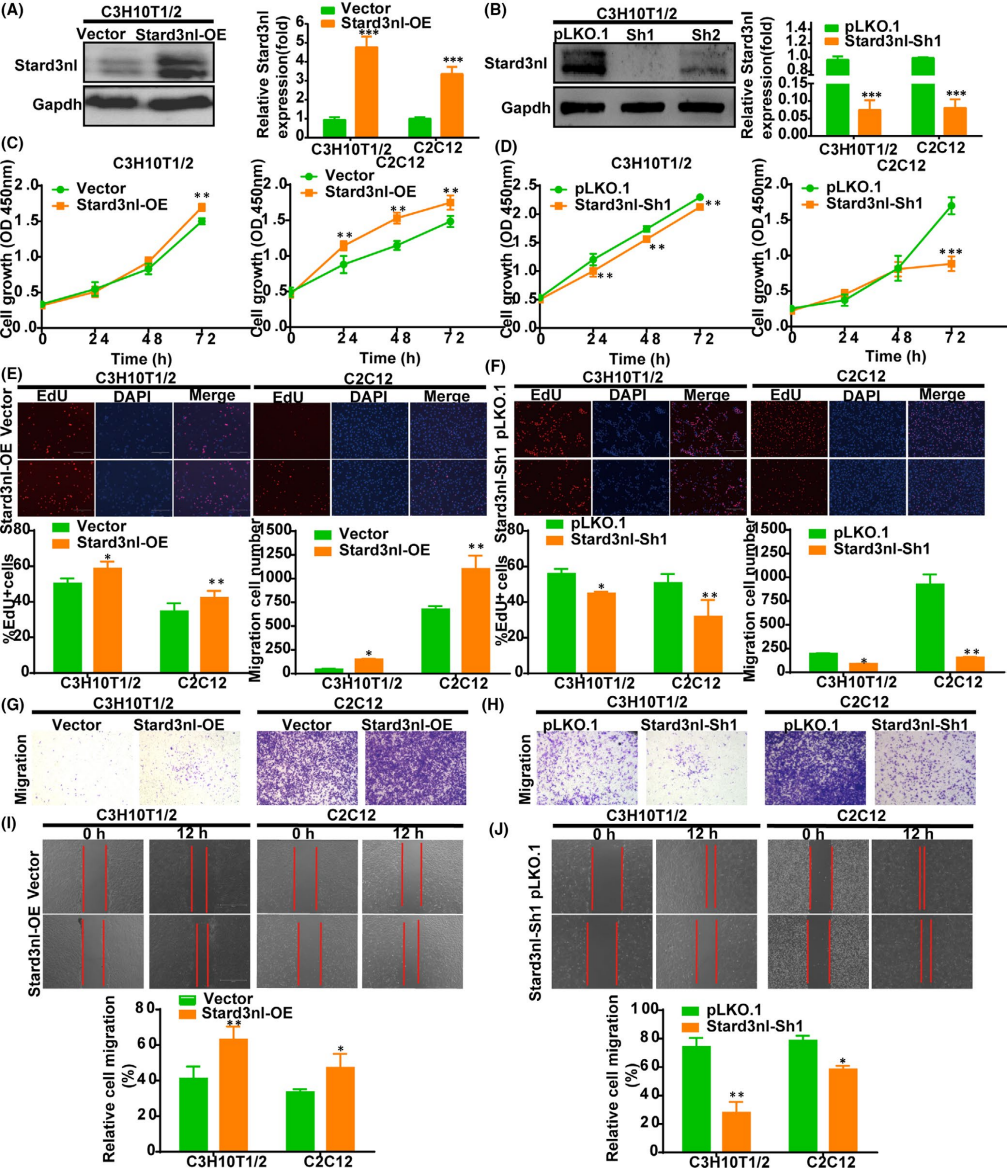
Enforced expression of Stard3nl promotes proliferation and cell migration of C3H10T1/2 and C2C12 cells. (A,B) Stard3nl protein expression and mRNA level by overexpression of Stard3nl and knockdown of Stard3nl in stably transfected cells. (C,D) The cell viability for overexpression of Stard3nl and knockdown of Stard3nl on C3H10T1/2 and C2C12 cells by CCK‐8 assays. (E,F) The effect of overexpression of Stard3nl and knockdown of Stard3nl on cell proliferation by EdU assays. (G,H) Representative images of transwell assays of C3H10T1/2 and C2C12 cells migration for 24 h, and the number of migrated cells from the corresponding groups. (I,J) Representative images of wound‐healing assay for migration of C3H10T1/2 and C2C12 cells after 0 and 12 h, and the migration rate after 12 h. *p < 0.05, **p < 0.01, and ***p < 0.001

To further explore the role of Stard3nl in above process, thus, C3H10T1/2 and C2C12 cells with stable exogenous Stard3nl (Stard3nl‐OE) were generated successfully. RT‐qPCR and Western blot results confirmed the efficiency of overexpression of Stard3nl (Figure 2A). Next, we tested the effects of Stard3nl on the viability of C3H10T1/2 and C2C12 cells. Ectopic expression of Stard3nl predominantly primarily lead to promotion of the cell proliferation in C3H10T1/2 and C2C12 cells, as evidenced by CCK‐8. This pro‐proliferative role of Stard3nl was also verified through EdU assay (Figure 2C,E). Transwell and wound‐healing assays were performed in C3H10T1/2 and C2C12 cells for the purpose of investigating the role of Stard3nl on the regulation of cell migration. The number of migrating cells from Stard3nl overexpression cells was increased with significance after cell incubation for 24 h in transwell assay system (Figure 2G). The migration of Stard3nl overexpression cells was quicker according to the wound‐healing assays, presenting with a significantly enhanced gap closure, as compared to control cells (Figure 2I), suggesting that Stard3nl overexpression enhances cell migration of C3H10T1/2 and C2C12 cells.

As shown above, the findings have demonstrated that the gain of function of Stard3nl promoted proliferation and migration of C3H10T1/2 and C2C12 cells. Hence, it was anticipated that the loss of function of Stard3nl should produce the opposite results. Our subsequent experiments indeed supported this notion.

Short hairpin RNA (shRNA) using a lentivirus plasmid was adopted for the knockdown (KD) of Stard3nl expression for the purpose of further evaluating the function of Stard3nl in osteogenic differentiation in mouse C3H10T1/2 and C2C12 cells, which then differentiate into osteoblasts. We utilized two specific shRNAs targeting Stard3nl, but Stard3nl‐sh1 effectively reduced Stard3nl mRNA and protein levels before BMP2 stimulation in C3H10T1/2 and C2C12 cells (Figure 2B). Conversely, evidence from CCK‐8 and Edu assays showed that depletion of Stard3nl in C3H10T1/2 and C2C12 cells suppressed cell proliferation (Figure 2D,F). Similarly, transwell migration and wound‐healing assay demonstrated that knockdown of Stard3nl distinctly reduced cell mobility of C3H10T1/2 and C2C12 cells (Figure 2H,J).

### 3.3 Ectopic expression of Stard3nl inhibits osteogenic differentiation of C3H10T1/2 and C2C12 cells

**FIGURE 3:**
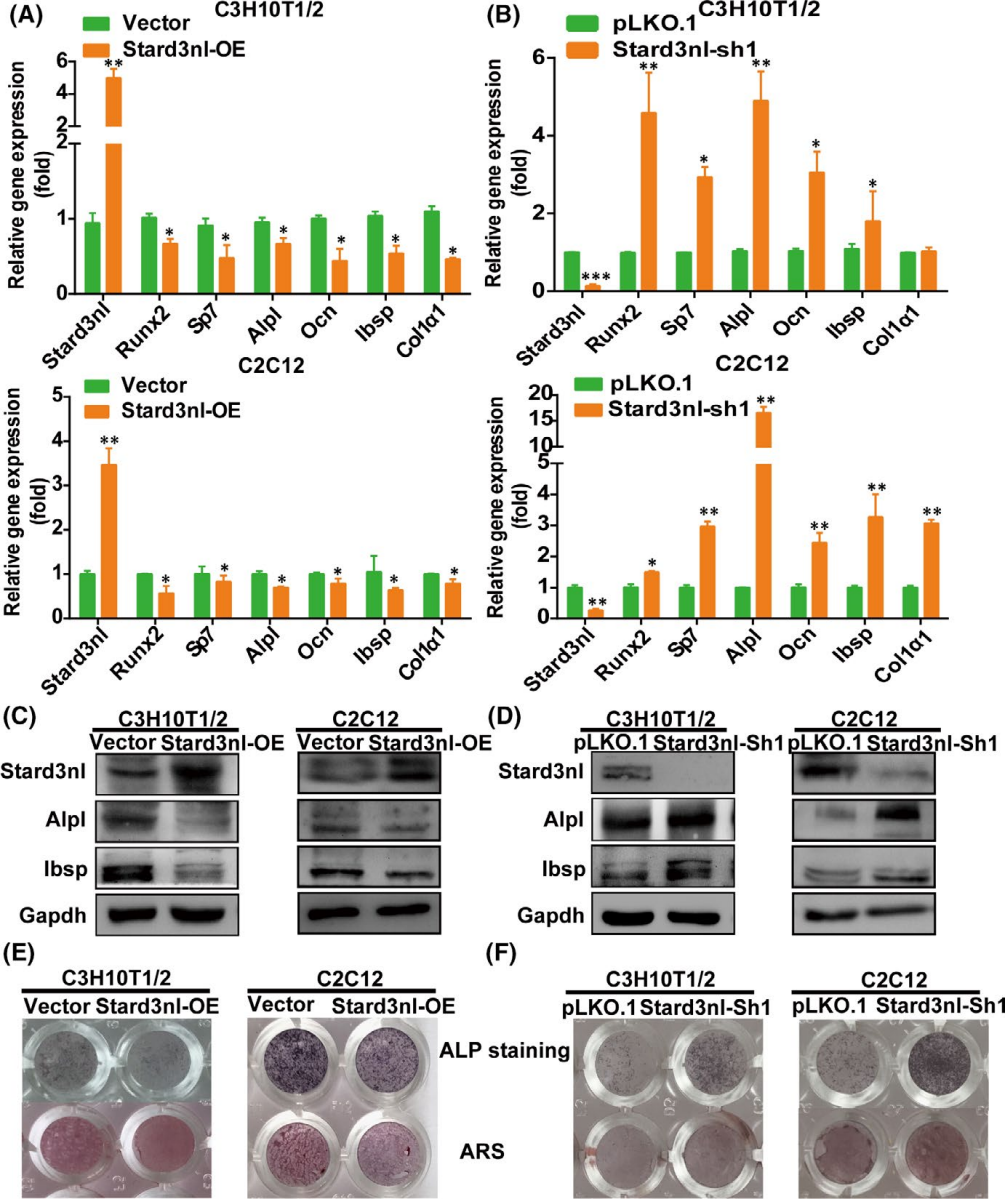
Overexpression of Stard3nl inhibits osteogenic differentiation of C3H10T1/2 and C2C12 cells. (A,B) RT‐qPCR analysis of Stard3nl and osteoblast markers after 48 h of culture normalized relative to Gapdh mRNA in stably transfected cells. (C,D) Western blot analysis of Stard3nl and osteoblast markers after 48 h of culture normalized relative to Gapdh in stably transfected cells. (E,F) ALP staining and ARS after 4–7 days of culture with BMP2 in stably transfected cells. *p < 0.05 and **p < 0.01

The above findings propose the upregulation of Stard3nl in patients with low bone mass and the potential of Stard3nl as a possible regulator in the osteogenic differentiation. Accordingly, osteoblast‐specific genes (Runx2, Sp7, Alpl, Ocn, Ibsp, Col1α1) were detected and showed decreased expression in Stard3nl‐transduced cells in comparison with the control group (Figure 3A,C). Being consistent with changes of their mRNA and protein levels, overexpression of Stard3nl in C3H10T1/2 and C2C12 cells leads to diminished osteogenic potential with slightly stained and less mineralized nodule formation after BMP2 stimulation, as revealed by ALP staining and ARS (Figure 3E).

Given the above gain‐of‐function results, the loss‐of‐function experiments were further conducted to confirm the function of Stard3nl. Relative to the control cells, mRNA levels and protein expressions of osteoblast marker genes were correspondingly elevated in Stard3nl KD cells during subsequent osteogenic differentiation (Figure 3B,D). Consistently, decreased expression of Stard3nl in C3H10T1/2 and C2C12 cells resulted in strengthened osteogenic potential with increased mineralization after BMP2 stimulation, as indicated by ALP staining and ARS (Figure 3F). The above findings confirmed the role of Stard3nl being a negative modulator in the regulation of osteogenic differentiation in C3H10T1/2 and C2C12 cells.

### 3.4 Stard3nl inhibits osteogenic differentiation via Wnt/β‐catenin signalling by transcriptomic analysis

**FIGURE 4:**
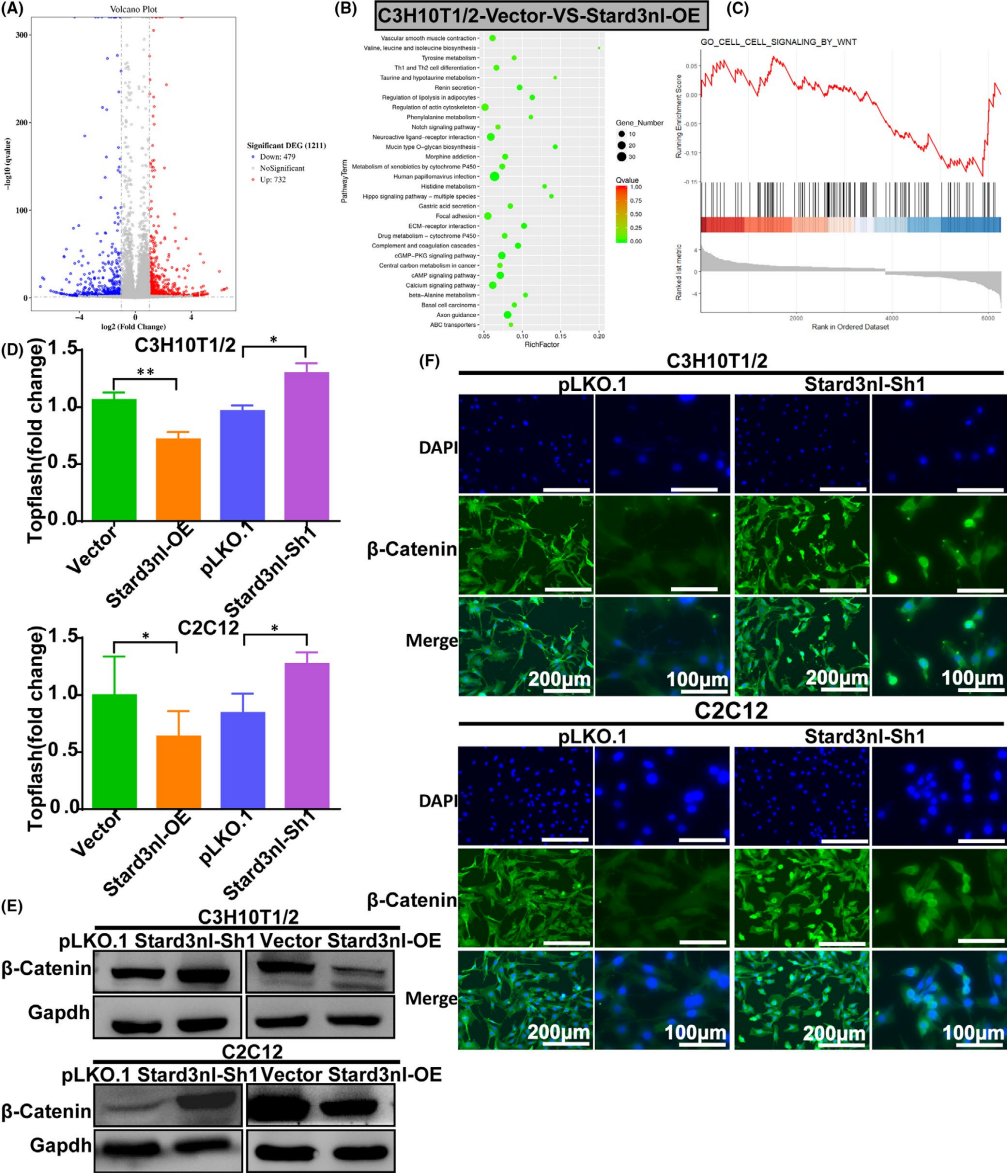
Stard3nl inhibits Wnt/β‐catenin signalling. (A) A volcano plot of RNA‐seq analysis of DEGs in C3H10T1/2 cells with enforced expression of Stard3nl. (B) Enrichment analysis of the KEGG pathway. The size and colour of the dots represent the number of enriched genes and the adjusted *p*‐values, respectively. (C) Gene set enrichment analysis (GSEA) was used to analyse the distribution of DEGs in Wnt signalling. (D) TOPFlah of mouse C3H10T1/2 and C2C12 cells expressing different levels of Stard3nl. (E) Western blot of β‐catenin levels in stably transfected cells. (F) Immunofluorescence of β‐catenin nuclear translocation on Stard3nl KD cells. *p < 0.05 and **p < 0.01

To gain insight into the underlying mechanism by which Stard3nl leads to suppression in osteogenic differentiation of C3H10T1/2 and C2C12 cells, we performed the transcriptomic analysis for screening of genes with altered expression levels induced by Stard3nl overexpression through RNA‐seq. The influence of Stard3nl overexpression was found of significance in the expression levels of 1211 genes compared to control in C3H10T1/2 cells; 732 genes were upregulated, and 479 genes were downregulated in Stard3nl overexpressed C3H10T1/2 cells (Figure 4A). Stard3nl regulated genes showed a strong relationship with cAMP signalling pathways and calcium signalling pathways evidenced by the results from KEGG pathway analysis of differentially expressed genes (DEGs) in C3H10T1/2 cells (Figure 4B). Next, Wnt signalling was implicated in Stard3nl regulated genes in C3H10T1/2 cells, by employing gene set enrichment analysis (GSEA) (Figure 4C). Moreover, β‐catenin/TCF/LEF transcriptional activity decreased by half in Stard3nl overexpression cells, while it increased 1.5 times in Stard3nl KD cells showed by the TOPFlash assay (Figure 4D). These results were further confirmed by Western blot analysis for comparison of total β‐catenin across the groups. The enforced expression of Stard3nl reduced the protein level of the total β‐catenin in C3H10T1/2 and C2C12 cells as shown in Figure 4E. Consistently, the overlap between DAPI and β‐catenin was enhanced in Stard3nl KD cells, suggesting that the nuclear translocation of β‐catenin was significantly increased in Stard3nl KD cells (Figure 4F).

### 3.5 Stard3nl inhibits Wnt/β‐catenin signalling via binding with Anxa2

**FIGURE 5:**
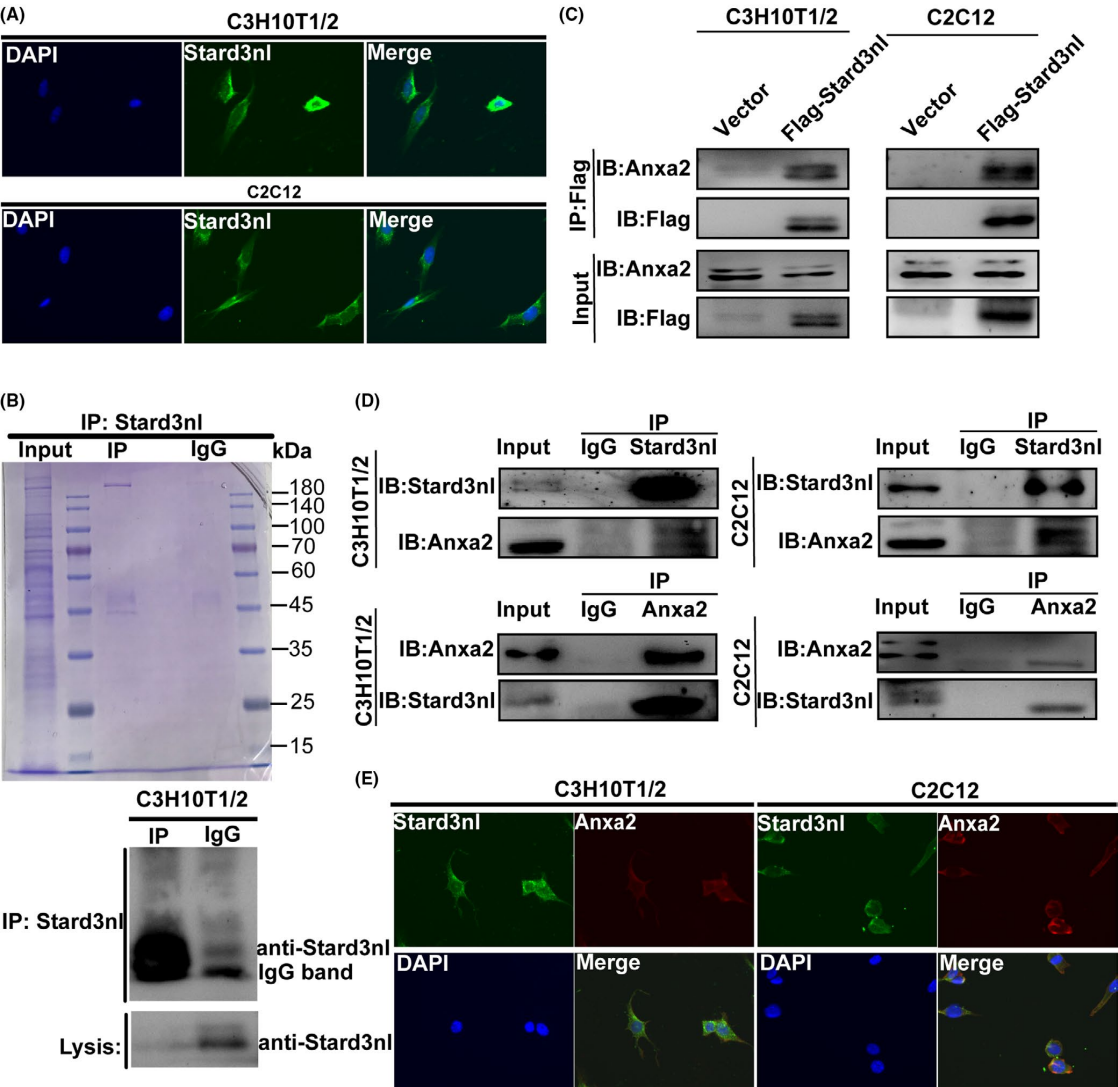
Stard3nl binds with Anxa2. (A) Representative images of the localization of Stard3nl (Green), mainly in the cytoplasm of both C3H10T/2 and C2C12 cells. DAPI was used for nuclei staining. (B) A Coomassie blue staining of purification of Stard3nl interactome complex (left panel). C3H10T1/2 cells were subjected to immunoprecipitation using anti‐Stard3nl antibody. (C) Co‐immunoprecipitation detected the bind of exogenous Stard3nl and Anxa2 in stably transfected cells using anti‐Flag antibody. (D) Coimmunoprecipitation detected the bind of endogenous Stard3nl and Anxa2 in both C3H10T/2 and C2C12 cells using anti‐Stard3nl or anti‐Anxa2 antibody with IgG as control. (E) Representative images showed that colocalization of Stard3nl (Green) and Anxa2 (Red) was observed mainly in the cytoplasm of both C3H10T/2 and C2C12 cells. Nuclei were stained with DAPI

As shown in Figure 5A, the localization of Stard3nl was primarily in the cytoplasm of both C3H10T/2 and C2C12 cells. With the intention to figure out the molecular that bind with Stard3nl and facilitate its effect in osteogenic differentiation, we performed immunoprecipitation of Stard3nl in C3H10T1/2 cells and mass spectrometry analysis for the identification of binding partner (Figure 5B). From the peptide sequencing, a total of 88 proteins were identified and Anxa2 appear to be one of the top‐ranking molecular that could bind with Stard3nl. In agreement with this finding, a previous study also demonstrated that Anxa2 could promote the β‐catenin nuclear translocation. This association between Stard3nl and Anxa2 in C3H10T1/2 and C2C12 cells was confirmed both endogenously and exogenously by co‐immunoprecipitation (co‐IP) experiments by utilizing anti‐Stard3nl or anti‐Anxa2 or anti‐Flag‐tagged antibodies (Figure 5C,D). Colocalization of Stard3nl and Anxa2 in the cytoplasm was evidenced by immunofluorescence staining of C3H10T1/2 and C2C12 cells (Figure 5E). Given these results above, we next investigated the possible mechanism concerning Stard3nl. Thus, Stard3nl‐overexpressing C3H10T1/2 and C2C12 cells with stable exogenous Anxa2 (Anxa2‐OE) were generated successfully, as confirmed by RT‐qPCR and Western blot (Figure S1A,B). Overexpression of Anxa2 reversed the diminished Runx2 protein levels and ALP staining in Stard3nl‐OE cells (Figure S1B,C). In addition, the TOPFlash assay and the nuclear translocation of β‐catenin also confirmed that overexpression of Anxa2 restored the decrease in the Wnt/β‐catenin signalling in the Stard3nl‐OE cells, indicating that Anxa2 is an integral part of the Stard3nl‐mediated Wnt/β‐catenin signalling involved in osteogenic differentiation (Figure S1D,E).

### 3.6 AAV9‐mediated silencing of Stard3nl prevented bone loss in OVX‐induced osteoporosis

**FIGURE 6:**
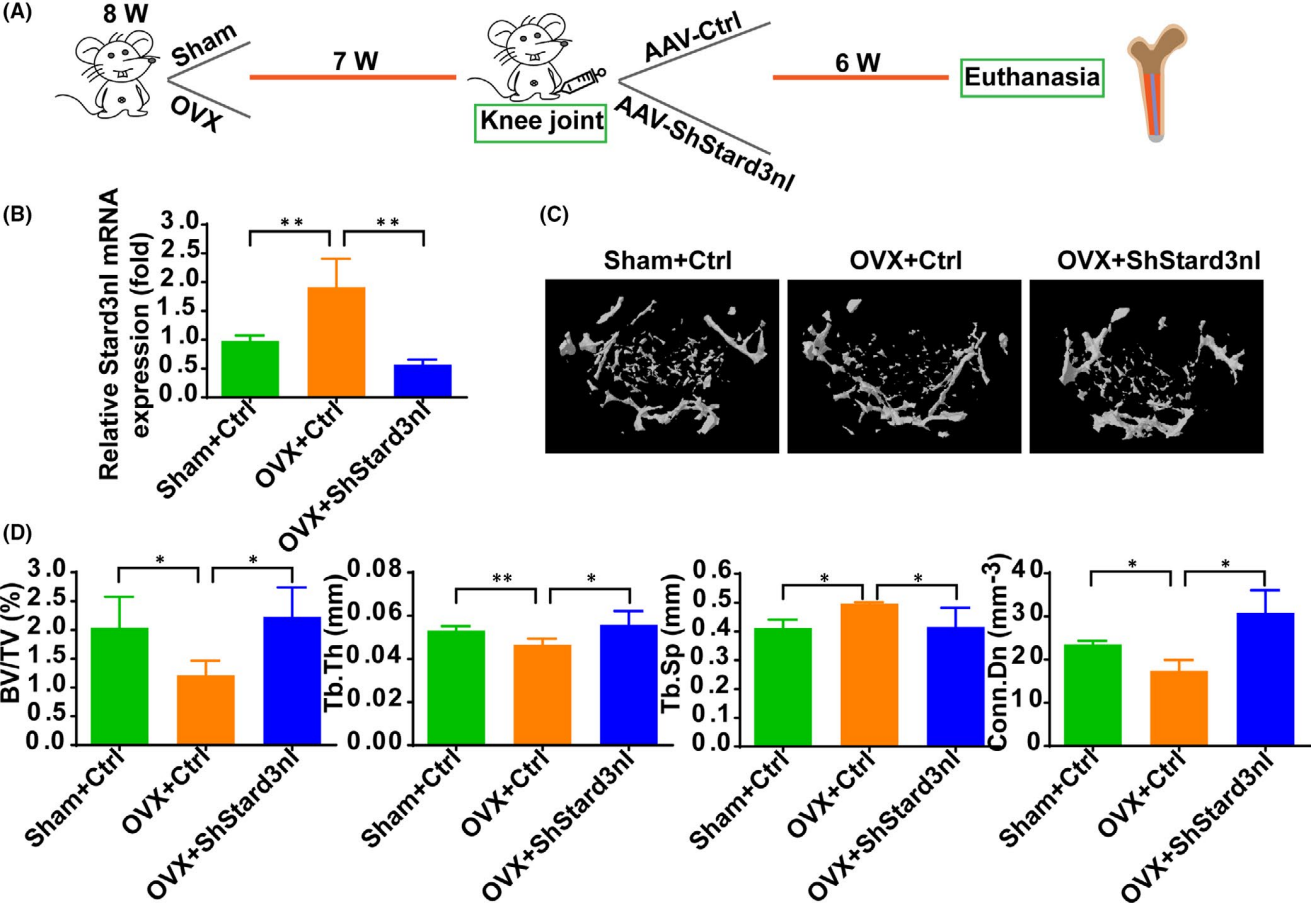
Knockdown of Stard3nl by local administrated AAV alleviates bone loss in OVX‐induced osteoporosis. (A) Schematic diagram of the study and the timeline of local administration of AAV‐Ctrl or AAV‐ShStard3nl into knee joints into OVX mice. (B) The mRNA level of Stard3nl in tibial bone. (C) Representative 3D reconstruction of femoral trabecular bone mass by microCT from indicated groups of mice. (D) Relative quantification of analysis: BV/TV, bone volume/ tissue volume (%); Tb.Th, trabecular thickness (mm); Tb.Sp, trabecular spacing (mm); Conn.Dn, connectivity density (mm‐3). *n* = 5 mice in each group. *p < 0.05 and **p < 0.01

**FIGURE 7:**
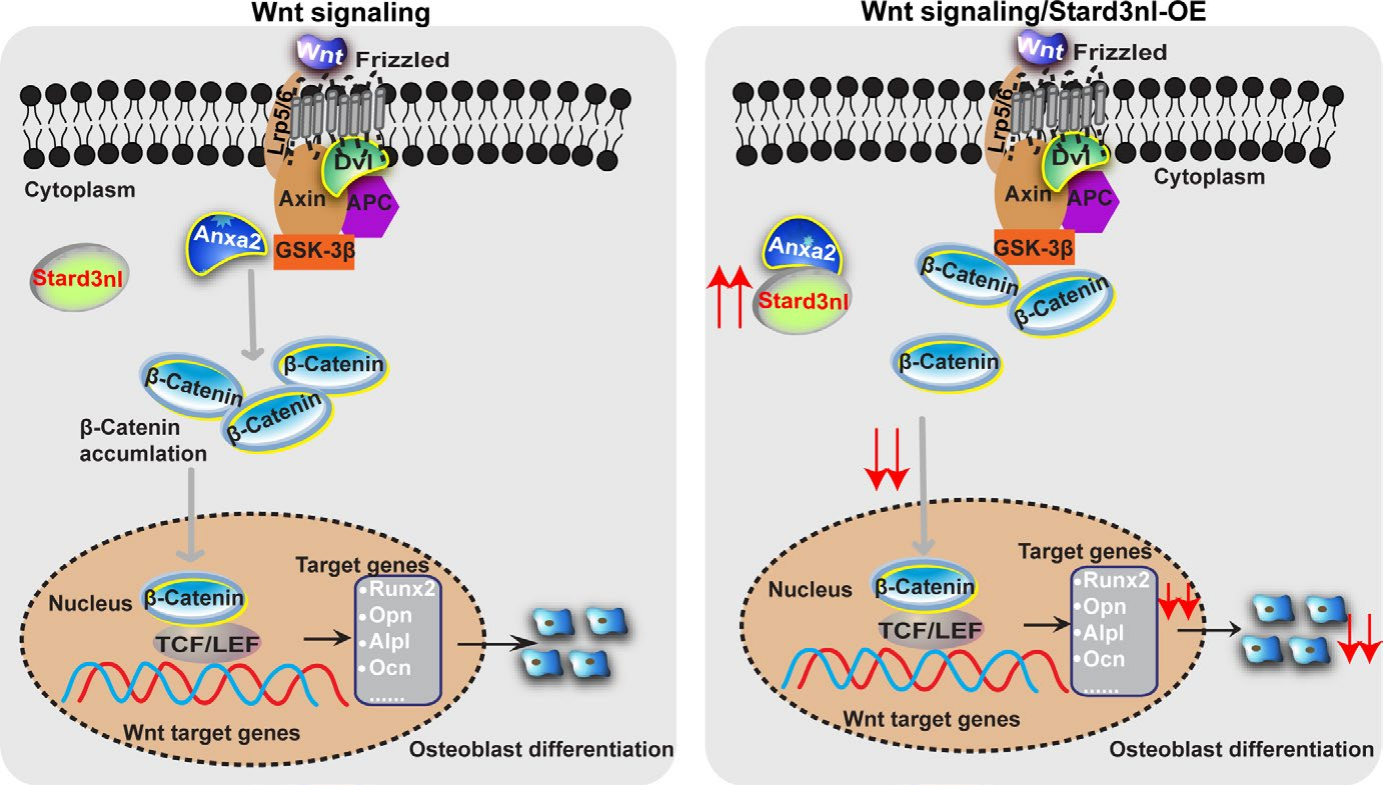
Schematic diagram representing the regulation of Stard3nl in osteogenic differentiation. Normally, Anxa2 promotes β‐catenin nuclear translocation, activating Wnt/β‐catenin signalling and inducing further stimulation of osteoblast marker genes for osteogenic differentiation. Upon upregulation of Stard3nl, Stard3nl binds with Anxa2, inhibiting β‐catenin expression and the nuclear translocation of β‐catenin, thus resulting into inactivation in Wnt/β‐catenin signalling and suppression of downstream osteogenesis

Considering that inhibition of Stard3nl promoted the osteoblast differentiation, we asked whether inhibition of Stard3nl in vivo could alleviate bone loss in osteoporosis. Thus, sham or OVX surgery was conducted on 2‐month‐old female mice to mimic models for osteoporosis. At seven weeks post‐OVX surgery, AAV9‐Ctrl or AAV9‐ShStard3nl was delivered via injecting into knee joints (Figure 6A). Six weeks following treatment, femurs transduced by AAV9‐ShStard3nl displayed efficient transduction by GFP expression, leading to ~50% reduction of Stard3nl mRNA and protein levels compared to AAV9‐Ctrl delivery (Figure 6B, Figure S2A,B). As expected, bone loss phenotype was partly reversed in the femur of OVX‐induced osteoporotic mice after AAV9‐ShStard3nl administration, as shown by reconstructed Micro‐CT 3D image, trabecular BV/TV, thickness (Tb. Th), separation (Tb. Sp)and connectivity density (Conn. Dn) (Figure 6C,D). Together, these results attest to the marked therapeutic effect of local administration of AAV9‐ShStard3nl in OVX‐induced osteoporosis, compared with local injection of AAV9‐Ctrl. Collectively these findings suggest that Stard3nl binds Anxa2, leading to the inactivation of Wnt/β‐catenin signalling and inducing further inhibition of osteogenic differentiation and bone formation (Figure 7).

## 4 DISCUSSION

Despite the fact that extensive studies on the regulatory roles of Stard3nl in bone development have been proposed based on GWAS results from patients with osteoporosis,^13^, ^14^, ^15^ how Stard3nl functions in modulating osteoblasts and associated bone formation remain obscure. In this paper, we substantiate that Stard3nl serves as a negative modulator in the regulation of osteogenic differentiation in vitro. Stard3nl deficiency in C3H10T1/2 and C2C12 cells resulted in enhanced BMP2‐induced osteoblastogenesis, while enforced expression of Stard3nl leads to less ALP activity and mineralization in C3H10T1/2 and C2C12 cells. Furthermore, silencing of Stard3nl in vivo reversed bone loss in OVX‐induced osteoporosis. Therefore, our data authenticate a novel negative regulatory role of Stard3nl in diminishing osteoblast differentiation and bone formation.

Stard3nl was initially identified as a Stard3 homologue anchored in the late endosome. Due to its MENTAL domain, the ubiquitously expressed Stard3nl functioned in maintaining cholesterol at the late‐endosomal membranes before its transportation to the cytoplasmic acceptors.^6^, ^21^ Like LTP‐binding cholesterols, STARD3, exchanging cholesterol between endosomes and the endoplasmic reticulum, STARD3NL might transport cholesterol within ER–endosome contacts.^11^ Furthermore, SNPs in the STARD3NL loci have strong association with BMD.^13^, ^14^, ^15^ Our results investigated that the role of STARD3NL at the STARD3NL loci in osteogenic differentiation may contribute to bone phenotypes. Our transcriptomic studies showed that Stard3nl was implicated in the Wnt signalling pathway in C3H10T1/2 cells. Activated canonical Wnt signalling not only facilitates osteoblast differentiation from MSCs but also restrains differentiation of which into osteoclast‐lineage cells or adipocytes.^22^, ^23^, ^24^, ^25^ For instance, USP7 and lncRNA DANCR inhibits Wnt/β‐catenin‐induced osteoblast differentiation in osteoporosis.^26^, ^27^ Two natural compounds, kaempferol and daphnetin, are found to stimulate differentiation of osteoblasts via activation of Wnt/β‐catenin signalling.^28^, ^29^ Wnt signalling appears to be a promising target pathway for development of novel bone anabolic drugs, such as the tested neutralizing antibodies against the sclerostin, a Wnt antagonist.^30^ In the absence of Wnt ligand signals, cytoplasmic β‐catenin protein, as a key factor in the Wnt signalling pathway, is routinely phosphorylated by glycogen synthase kinase 3 beta (GSK3β), and the phosphorylated β‐catenin accumulates in the cytoplasm and undergo degradation. Meanwhile, activation of the Wnt signalling pathway induces a cascade of biological events that eventually stabilizes β‐catenin and promotes its transnucleation by suppressing GSK3β kinase activity,^31^ promoting osteogenic differentiation with the elevated transcription activity of osteoblast‐specific genes, including Runx2, Alpl, Ocn and Ibsp.^32^ Suppression or overexpression of Stard3nl resulted into increased or restrained Wnt signalling activity in C3H10T1/2 and C2C12 cells, respectively. Our findings introduce Stard3nl as a new, negative regulator in Wnt signalling pathway in osteogenic differentiation.

However, the potential mechanism for crosstalk between Stard3nl and Wnt/β‐catenin remains obscure. Given that the localization of Stard3nl is mainly in the cytoplasm of both C3H10T1/2 and C2C12 cells, an LC‐MS study was performed to explore the possible mechanism, and Anxa2 emerged as the protein binding with Stard3nl. Anxa2, a 36 kD calcium‐mediated phospholipid‐binding protein, is the most studied member of annexins and is involved in multiple critical cellular processes, for instance, cell proliferation, cell adhesion, cell motility, angiogenesis and endocytosis.^33^ Previous research demonstrated the capability of ANXA2 in complexing with FOXQ1 to modulate β‐catenin expression in bone mesenchymal stem cells (BMSCs).^34^ lncRNA‐MUF binds to ANXA2, enhancing binding of which to GSK3β and hence inhibiting the formation of the GSK3β/β‐catenin complex.^35^ Our data also show that the suppressive role of Stard3nl is for its bind with Anxa2, which is crucial for the generation of a repressed state on the Wnt signalling pathway.

To test the therapeutic effect of silencing of Stard3nl in vivo, an OVX‐induced osteoporosis model is established and gene targeting by AAV vectors are introduced, which own a long‐term safety and efficacy in both preclinical and clinical studies.^36^ Previous studies demonstrated that a recombinant AAV9 was highly effective for transducing both osteoblast and osteoclast‐lineage cells in the bone. Importantly, rAAV9‐mediated silencing of Schnurri‐3 in osteoblasts or/and rAAV9‐mediated gene silencing (RANK and CTSK) in osteoclasts are both rAAV9‐based gene therapy for osteoporosis.^37^, ^38^ In addition, AAV‐mediated FOXO3a‐RNAi, AAV‐mediated MVP and AAV‐shIGSF23 with a local administration or a systemic delivery also protect mice from pathologic bone loss.^39^, ^40^, ^41^ Through this approach, we injected AAV‐ShStard3nl into knee joints of OVX mice, and a significant increase in bone mass was displayed, suggesting a novel AAV‐based gene therapy for osteoporosis.

Given that Stard3nl negatively regulates osteogenic differentiation, SNPs in the STARD3NL may have association with alterations in osteoblast activity that might play a role in the development of postmenopausal osteoporosis. Yet, further investigation is required. In conclusion, our research elucidates the mechanisms by which Stard3nl modulates osteogenic differentiation, substantiating that Stard3nl serves as a negative regulator of osteogenic differentiation, which is critical for bone remodelling under both physiological and pathological conditions.

## CONFLICT OF INTEREST

The authors report no conflict of interest.

## AUTHOR CONTRIBUTIONS

**Yuexin Xu:** Formal analysis (lead); Investigation (lead); Writing – original draft (lead). **Xiaogang Bao:** Investigation (supporting); Resources (lead); Validation (supporting). **Xiaoyun Chen:** Formal analysis (equal); Investigation (equal); Writing – review & editing (lead). **Peixuan Wu:** Formal analysis (supporting); Investigation (supporting); Validation (supporting). **Shiyu Chen:** Formal analysis (supporting); Investigation (supporting). **Bowen Zhang:** Formal analysis (supporting); Investigation (supporting). **Jing Ma:** Supervision (lead); Writing – review & editing (lead). **Guohua Xu:** Funding acquisition (supporting); Resources (lead); Supervision (lead). **Duan Ma:** Conceptualization (lead); Funding acquisition (equal); Writing – review & editing (supporting).

## Supporting information

Supplementary Material

## Data Availability

The data sets used and/or analysed during the current study are available from the corresponding author on reasonable request.
