# Supplementary material for: STARD3NL inhibits the osteogenic differentiation by inactivating the Wnt/β‐catenin pathway via binding to Annexin A2 in osteoporosis
Source: J Cell Mol Med. 2022 Jan 30;26(5):1643–55. doi: 10.1111/jcmm.17205 (PMC8899174; doi:10.1111/jcmm.17205)
Supplement: Supplementary file 1 — Supplementary Material [file JCMM-26-1643-s001.docx]

**
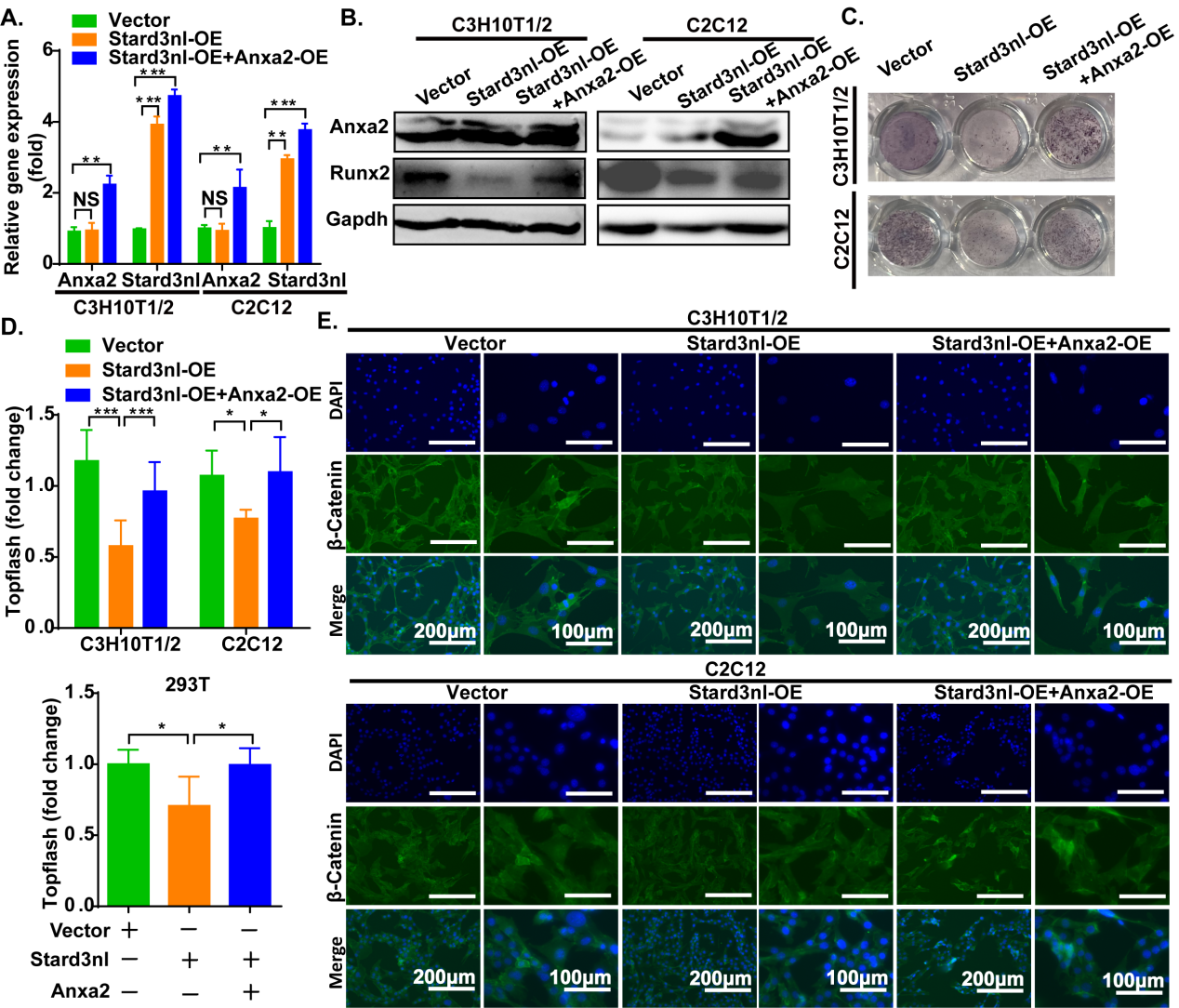
Supplemental Figure 1.** Anxa2 is an integral part of the Stard3nl-mediated Wnt/β-catenin signaling. A, Anxa2 and Stard3nl mRNA levels by overexpression of Anxa2 in Stard3nl-OE cells. B, Anxa2 and osteoblast marker Runx2 protein expression by overexpression of Anxa2 in Stard3nl-OE stably transfected cells. C, ALP staining showing ALP activity in Stard3nl-OE cells transduced with Anxa2 overexpression. D, The TOPFlash assay of C3H10T1/2, C2C12 and 293T cells expressing different levels of Stard3nl and Anxa2. E, Immunofluorescence of β-catenin nuclear translocation on Stard3nl-OE cells transduced with Anxa2 overexpression. *p < 0.05, **p < 0.01, and ***p <0.001.

**
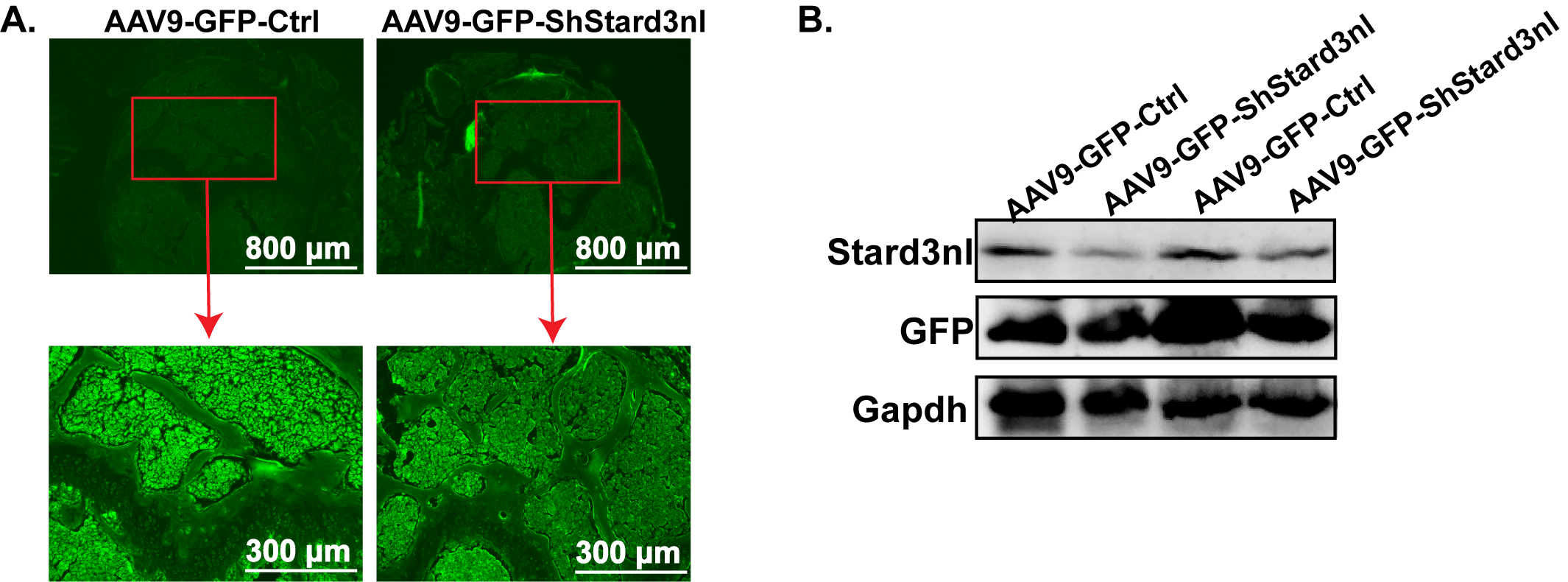
**

**Supplemental Figure 2.** GFP signal showing the successful AAV9 delivery in vivo. A, Green fluorenscence intensity of ferums injecting with AAV9-GFP-Ctrl or AAV9-GFP-ShStard3nl. B, Expression of GFP and Stard3nl in the ferum was assessed by western blot.
